# Preparation of Diosgenin-Functionalized Gold Nanoparticles: From Synthesis to Antitumor Activities

**DOI:** 10.3390/ijms26031088

**Published:** 2025-01-27

**Authors:** Elżbieta U. Stolarczyk, Weronika Strzempek, Magdalena Muszyńska, Marek Kubiszewski, Anna B. Witkowska, Kinga Trzcińska, Piotr Wojdasiewicz, Krzysztof Stolarczyk

**Affiliations:** 1 Spectrometric Methods Department, National Medicine Institute, 30/34 Chełmska Street, 00-725 Warsaw, Poland; e.stolarczyk@nil.gov.pl (E.U.S.); anna.witkowska@wum.edu.pl (A.B.W.); 2 Faculty of Chemistry, Jagiellonian University, 2 Gronostajowa Street, 30-387 Krakow, Poland; weronika.skuza.s@gmail.com; 3 Faculty of Chemistry, University of Warsaw, 1 Pasteura Street, 02-093 Warsaw, Poland; m.muszynska@chem.uw.edu.pl; 4 Analytical Research Section, Pharmaceutical Analysis Laboratory Łukasiewicz Research Network, Industrial Chemistry Institute, 8 Rydygiera Street, 01-793 Warsaw, Poland; marek.kubiszewski@ichp.lukasiewicz.gov.pl (M.K.); kinga.trzcinska@ichp.lukasiewicz.gov.pl (K.T.); 5 Department of Biophysics, Physiology and Pathophysiology, Faculty of Health Sciences, Medical University of Warsaw, Chałubińskiego 5, 02-004 Warsaw, Poland; piotr.wojdasiewicz@wum.edu.pl

**Keywords:** diosgenin, thiol derivatives, biological active compound, drug carriers, anticancer drugs, nanoparticles, cytotoxic study, cancer cells, SP-ICP-MS

## Abstract

Cancer ranks among the top causes of illness and death globally. Nanotechnology holds considerable promise for enhancing the effectiveness of therapeutic and diagnostic approaches in cancer treatment. Our study presents a promising strategy for applying thiocompound nanomedicine in cancer therapy. Our first study aimed to investigate the biological properties of a new compound thiodiosgenin (TDG)—a new derivative of diosgenin—a natural compound with known antioxidant and anticancer properties. Our current second study aimed to compare the therapeutic efficacy of a new diosgenin—functionalized gold nanoparticles—with its precursor on prostate cancer (DU-145) cell lines. Moreover, the safety of the new thio-derivative and new conjugates was tested against the human epithelial line PNT-2. New advanced analytical techniques were developed for the characterization of nanomaterials using methods such as SP-ICP-MS, UV-Vis, TEM, NMR, FT-IR ELS, and TGA. Our synthetic approach was based, on the one hand, on the ligand exchange of citrates to thiodiosgenin (TDG) on gold nanoparticles, and on the other hand, on the attachment of DG through an ester bond to the linker, which was 3-mercaptopropionic acid (MPA) on gold nanoparticles. Initial in vitro studies indicate that TDG shows greater cytotoxic effects on cancer cells but poses risks to normal prostate epithelial cells (PNT-2). It was demonstrated that all the conjugates produced exhibited significant cytotoxic effects against cancer cells while being less harmful to normal prostate epithelial cells (PNT-2) compared to TDG itself. All the obtained conjugates showed antitumor properties; however, for targeted transport, the system referred to as AuNPs-MPAm1-DG is promising, due to the size of the nanoparticles of 53 nm, zeta potential of -30 mV, and loading content of 27.6%. New methods for synthesizing conjugates with diosgenin were developed and optimized for medical applications. Advanced new analytical methodologies were developed to characterize new conjugates, particularly the use of SP-ICP-MS, to solve existing differences in the shape and morphology of the surface of new conjugates.

## 1. Introduction

Gold nanoparticles (AuNPs), due to their various surface characteristics and unique qualities, are widely exploited in nanotechnology. Gold nanoparticles (AuNPs) can be readily modified, which allows them to serve as a flexible platform for nanobiological constructs that include peptides [1,2], oligonucleotides [3], antibodies [4], proteins [5], and thiol compounds. Sulfur-containing compounds have been used as excellent ligands for binding to metal nanoparticles, especially to Au nanoparticles, due to the very strong interaction of sulfur nucleophiles with gold surface monolayers [6], and gold nanoparticles [7,8,9,10]. Organic thiols [11] stabilize the colloidal suspension and functionalize the gold surface through a Au–S covalent bond (40 kcal/mol^−1^) [12]. The application of thiol groups for the attachment of biologically active substances to gold nanoparticles is widely recognized and firmly established in the scientific literature [13,14]. The attachment of various compounds to gold nanoparticles (AuNPs) has the potential to alter their rheological properties, such as surface plasmon resonance, redox activity, electrical conductivity, etc. [15,16,17,18]. Therefore, they can result in notable signals in diagnostics and as a platform for therapeutic agents because of their enormous surface area. Conjugates of this type are recognized for enhancing the therapeutic effectiveness of numerous drugs by improving their water solubility, stability, and bioavailability, as well as facilitating direct drug delivery to the target receptor and increasing cell membrane permeability [19,20]. Furthermore, gold nanoparticles that are coated with specific proteins through a terminal sulfur atom have already been utilized in the fields of organogenesis [21] and clinical diagnostics [22,23].

The synthesis of gold nanoparticles has already been well established. Gold nanoparticles can be synthesized by the following primary methods: physical [24], chemical [25,26,27,28,29], biological, or by extracts of medicinal plants and microorganisms [1,30,31,32,33,34]. Gold nanoparticles can be prepared in various ways [35,36] including UV radiation, laser radiation, ultrasound, electrodeposition, or synthesis in solution using appropriate reducing compounds. The AuNPs can be synthesized in the presence of various compounds and systems: micelles, surfactants, membranes, amphiphilic compounds, and also in microorganisms. Their size is controlled by using the appropriate molar ratio of the AuCl_4_^−^ anion, stabilizer, and reductant. The temperature of the reaction system has a significant effect, as well as the rate of adding the reductant during synthesis [37]. The process of synthesizing nanoparticles from chloroauric acid has attracted significant attention due to the wide-ranging applications of these nanoparticles across various domains, including chemistry, photocatalysis, cancer treatment, and drug delivery. Additionally, their properties, which include antioxidant, antibacterial, and cytotoxic effects, further enhance their appeal in scientific research [38,39,40,41]. The application of nanoparticles across different domains is enabled by several factors, such as surface plasmon resonance, as well as the shape and size of the nanoparticles [42,43].

During chemical synthesis, the size, shape, and surface functionality can be controlled [25,26]. The basic method for synthesizing AuNPs by boiling hydrogen tetrachloroaurate (HAuCl_4_) with sodium citrate in an aqueous solution was described in 1951 [44]. Citrates perform stabilizing functions. Particle size can be adjusted by modifying the ratio of gold to citrate. In this way, spherical AuNPs with diameters of 10 to 20 nm as well as larger AuNPs (e.g., 100 nm) can be obtained. The Turkevich–Frens methods, which are conducted “in situ”, remain fundamental pathways for synthesis, utilizing citrate. These methods have been enhanced and expanded to include drugs and macromolecules, offering significant biomedical potential for optical and theranostic applications [45]. Dinkel and co-workers [46] showed that citrates are easily exchanged for various molecules with anchoring groups such as thiolates, amines, phosphine, carboxylates, etc. Citrate-stabilized nanoparticles may undergo irreversible aggregation when modified with thiolate ligands. This process requires appropriate optimization [47,48,49]. The synthesis of alkane thiol-stabilized AuNPs [47] can be carried out by a two-phase reduction, using tetraoctylammonium bromide as the phase transfer reagent and sodium borohydride (NaBH_4_) as the reducing agent [47]. By varying the ratio of gold to thiol, the reduction rate, and the reaction temperature, nanoparticles of different sizes can be obtained [37,50]. Usually, as a result of increasing the molar ratio of alkanethiol to gold salt, and lowering the temperature, particles with smaller diameters and monodisperse are obtained. In the research, it is crucial to select the appropriate concentration of the reducing agent, e.g., NaBH_4_, and the time of its addition. It turns out that increasing or decreasing the concentration of NaBH_4_ to gold ions or stabilizers can cause both an increase and a decrease in the diameter of the resulting nanoparticles [51,52]. The synergistic effect of thiol and gold generated strong van der Waals bonds and attraction between adjacent ligands, leading to superior stability of these alkanethiol-protected AuNPs relative to many other types of AuNPs [1,50]. The “in situ” Brust–Schiffrin methods remain the primary synthetic approaches using thiolate ligands, which have been refined and expanded to include drugs and macromolecules with significant biomedical potential for optical and theranostic applications [45]. Sperling and co-workers, preparing gold nanoparticles using NaBH_4_, observed an increase in the size of gold nanoparticles with a decrease in the concentration of NaBH_4_, as well as the formation of two distributions of particles with small and large diameters with a further decrease in the concentration of NaBH_4_ [51]. Lin and co-workers also synthesized gold nanoparticles using NaBH_4_ [52]. They observed the formation of nanoparticles with sizes from 1.7 to 8.2 nm, with an increase in the concentration of NaBH_4_. Too much NaBH_4_ can cause the formation of a lot of small nuclei in a short time, which can later aggregate in an uncontrolled manner, resulting in a high polydispersity of the sample or its complete aggregation [53]. Therefore, it is important during the optimization of the synthesis not only to choose the appropriate ratio of chloroauric (III) acid to ligand but also to choose the appropriate concentration of NaBH_4_.

**Figure 1:**
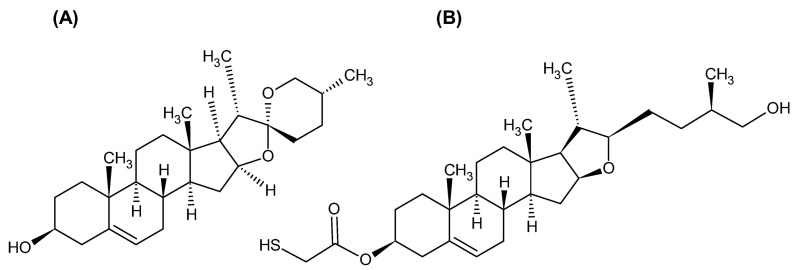
(**A**) Diosgenin (DG) structure; (**B**) thiodiosgenin (TDG) structure.

Diosgenin, (25R)-spirost-5-en-3β-Ol (Figure 1A), is a naturally occurring steroidal sapogenin that can be extracted from the seeds of fenugreek (*Trigonella foenum-graecum*) and the roots of wild yam (*Dioscorea villosa*). It is a key active component commonly found in medicinal plants such as *Rhizoma polgonita* and *Dioscorea rhizome* [54,55]. Furthermore, it acts as the precursor for the biosynthesis of steroid hormones [56] and an important natural pharmaceutical active ingredient [57]. Several pharmacological functions of DG have been previously reported [55,58,59]. Diosgenin, a bioactive compound, is utilized in the treatment of various medical conditions, including cancer, due to its diverse biological activities [60]. Preclinical research has demonstrated that diosgenin effectively inhibits the proliferation and growth of tumor cells, promotes differentiation and autophagy, induces apoptosis, prevents the metastasis and invasion of tumor cells, disrupts the cell cycle, modulates immune responses, and enhances gut microbiome health [61].

To enhance the biological effectiveness and bioavailability of diosgenin, researchers are concentrating on creating nano-drug carriers, combination therapies, and derivatives of diosgenin [61,62,63]. Limited data are available that focus on the synthesis of diosgenin nanoconjugates for their enhanced anticancer activities and improved delivery. Drug delivery systems (DDSs) such as nano-DDS-carrying therapeutic agents are emerging and being used in treating cancer with diosgenin, including mainly nanohydrogel and nanofibrillar structures [61]. Additionally, innovative polymer prodrugs were developed, including nanoparticles composed of diosgenin and methoxy-polyethylene glycol-4-formylbenzoate (mPEG-CBA), as well as an octagonal polyethylene glycol-diosgenin conjugate (8armPEGDGN/HCPT polymer nanoparticles), to enhance the therapeutic effectiveness of diosgenin in targeting tumor cells [64,65].

Silver nanoparticles combined with diosgenin can enhance the treatment of ascites lymphoma by modulating blood parameters and reducing oxidative stress [66]. Iron oxide nanoparticles (IONPs) functionalized with diosgenin can be used as magnetic nanocarriers with antiproliferative properties, migration inhibition, and apoptosis-inducing properties against breast cancer [67]. Diosgenin-conjugated gold nanoparticles (AuNPs) were synthesized using an algal extract as a reducing agent and tested on the colorectal cancer cell line HCT116 and the breast cancer cell line HCC1954 [68]. The results indicated that the extract-conjugated AuNPs exhibited anticancer activities that were approximately 18 times more potent than AuNPs alone. Additionally, diosgenin itself showed minimal anticancer effects on both cell lines.

The primary approaches for generating diosgenin derivatives with anticancer properties involve either traditional organic synthesis techniques or the use of biocatalysts [69,70]. A set of diosgenin derivatives substituted with various glucosides, amino acids, and esters is also known [71,72,73,74,75,76]. A method for introducing a thiol group into the structure of diosgenin (compound No. 20) was also developed [23]. The thiol group is an excellent ligand for binding to metal nanoparticles, particularly Au-nanoparticles, due to the very strong interaction of sulfur nucleophiles with gold. The structural formula of thiol derivatives of diosgenin (TDG) is presented in Figure 1B. To improve the biological activity and bioavailability of drugs, thiol derivatives of drugs attached to nanoparticles by a thiol linker can be considered.

Considering all this, in the present work, we focused on the anticancer effect of the gold nanoparticle systems with diosgenin. We also investigated the antitumor activity of structurally modified diosgenin with a thiol group. This will provide new information for the development of diosgenin with higher anticancer activity and lower toxicity. To comprehend and enhance the characteristics of the interaction, the colloidal gold nanoparticle (AuNP) system was analyzed in both its solid state and suspended form, before and following DG loading. In particular, UV–Visible spectroscopy, Nuclear Magnetic Resonance (NMR), Fourier-transform infrared (FTIR) spectroscopy, Electrophoretic Light Scattering (ELS), the ζ-potential, Transmission Electron Microscopy (TEM), single-particle (SP)-ICP-MS, and Thermogravimetry (TGA) were employed for characterization of the loaded and free system.

Drug delivery systems (DDSs) for cancer treatment include various nanostructures such as metal nanoparticles or metal oxide [77,78,79,80,81]. Wen et al. prepared a biodegradable amphiphilic triblock copolymer composed of monomethoxy polyethylene glycol, polycaprolactone, and poly-L-arginine (mPEG−PCL-PArg) and self-assembled with cisplatin (CDDP) to form NPs@CDDP (drug-loaded nanocapsules) [82]. These materials exhibit cytotoxicity towards cancer cells. The most popular nanoparticles are gold nanoparticles. The preliminary studies described in this paper are relevant to the selection of an appropriate route for the synthesis of new nanogold conjugates and their anticancer potential. However, it should not be forgotten that the new smarter systems are ultimately aimed at advancing in vivo functionality. The study of nano-bio-interactions and desirable functionality at the tissue, cellular, and molecular levels is very important [83]. The stealth effect plays a central role in improving pharmacokinetics such as blood circulation, biodistribution, and tissue targeting [84]. Smart systems are the future of nanomedicine.

## 2. Results and Discussion

### 2.1. Chemistry and Characterization

**Figure 2:**
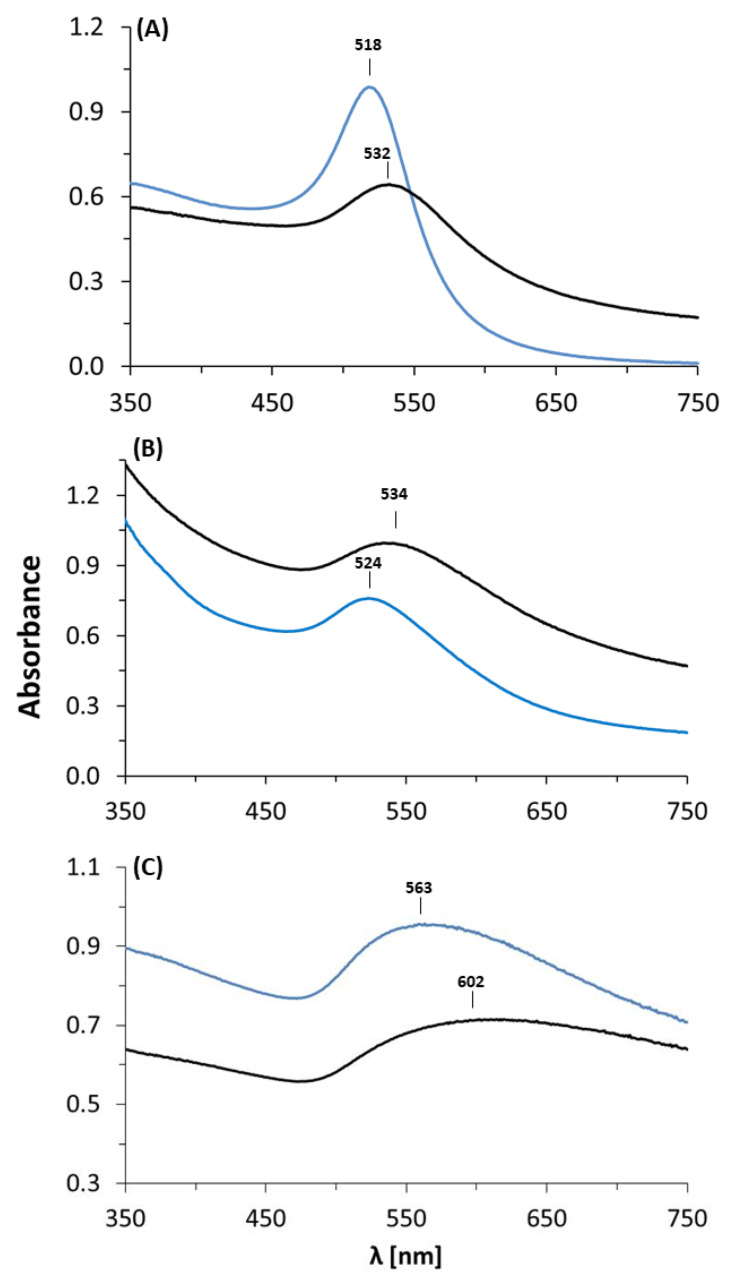
UV-Vis spectra of (**A**) AuNPs-citr (blue line) and AuNPs-TDG (black line), (**B**) AuNPs-MPAm1 (blue line) and AuNPs-MPAm1-DG (black line), and (**C**) AuNPs-MPAm1 (blue line) and AuNPs-MPAm1-DG (black line).

Au(III) in HAuCl_4_ has a high reduction potential and can be reduced by appropriate compounds. In this study, we synthesized gold nanoparticles (AuNPs) with citrates (citr), as previously described in Section 3.4.2, and 3-mercaptoproponic acid (MPA), as previously described in Section 3.4.1, which facilitates the donation of electrons to the metal, reducing Au^3+^. The effective binding and interaction between substrates and gold nanoparticles were additionally confirmed through UV–Visible spectroscopy, as illustrated in Figure 2. The formation of AuNPs-citr and AuNPs-MPA was confirmed by the color change and the presence of the localized surface plasmon resonance (LSPR) band, which occurs on the surface of some metals at the nanometer scale. The absorption intensity and peak position of LSPR spectra strongly depend on the morphology, size, shape, and accumulation of AuNPs. The spectral analysis distinctly indicates that the surface modification of the nanoparticles occurred, as evidenced by a notable redshift in the localized surface plasmon resonance (LSPR) position when compared to citrate-stabilized nanoparticles. UV-Vis spectroscopy demonstrated a peak at 518 nm for AuNPs-citr. This peak shifted to 532 nm due to citrate exchange for TDG, which simultaneously confirmed the effectiveness of the exchange (Figure 2A black line). The 524 nm peak was observed for AuNPs-MPAm1 (Figure 2B blue line), which indicated small nanoparticles compared to AuNPs-MPAm2 (Figure 2C blue line), showing a broad peak at 563 nm (Figure 2C blue line). Therefore, it was intriguing to further characterize the AuNPs-MPAm1 and AuNPs-MPAm2 samples in order to explain the differences in UV-Vis spectra.

**Figure 3:**
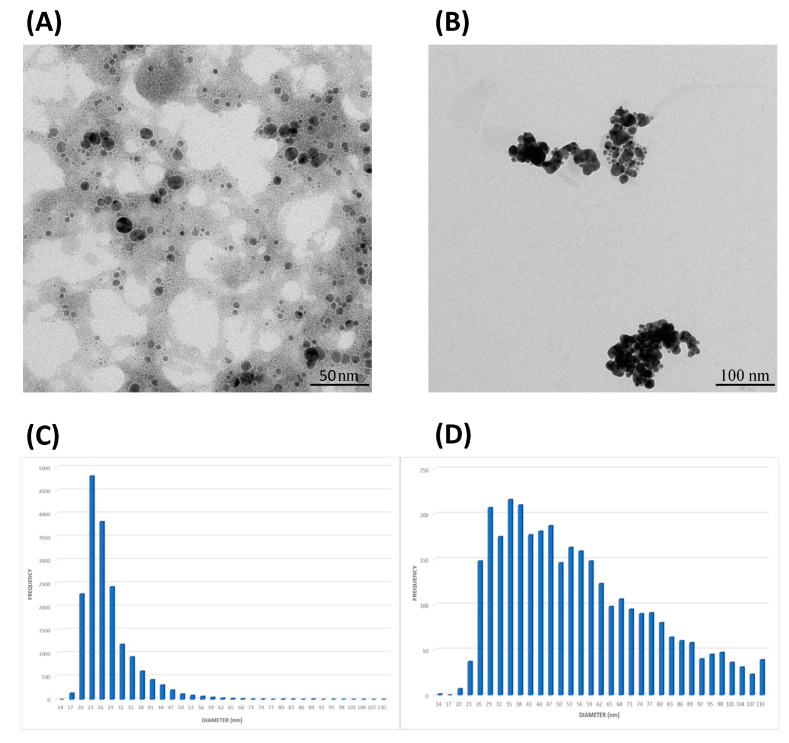
The morphologies of the synthesized AuNP-MPA. Comparison of characteristics obtained in TEM and SP-ICP-MS. (**A**,**B**) TEM image of AuNP-MPAm1 and AuNP-MPAm2, respectively (**C**,**D**) SP-ICP-MS particle size distribution of AuNP-MPAm1 and AuNP-MPAm2, respectively.

The morphologies and diameter distributions of AuNPs formed by MPA were investigated using the Transmission Electron Microscopy (TEM) analysis and Single-Particle–Inductively Coupled Plasma–Mass Spectrometry (SP-ICP-MS). Figure 3A shows AuNP-MPAm1 with mostly spherical shapes. Figure 3C shows the particle size histograms of synthesized AuNP-MPAm1 by SP-ICP-MS. The average size, measured by ICP-MS, was 27.8 nm. Figure 3B,D show AuNP-MPAm2 with the nanostructures exhibiting a spherical shape with a polycrystalline structure, suggesting the formation of agglomerates. The average size, measured by SP-ICP-MS, was 58.8 nm but with a histogram indicating a polycrystalline structure.

**Figure 4:**
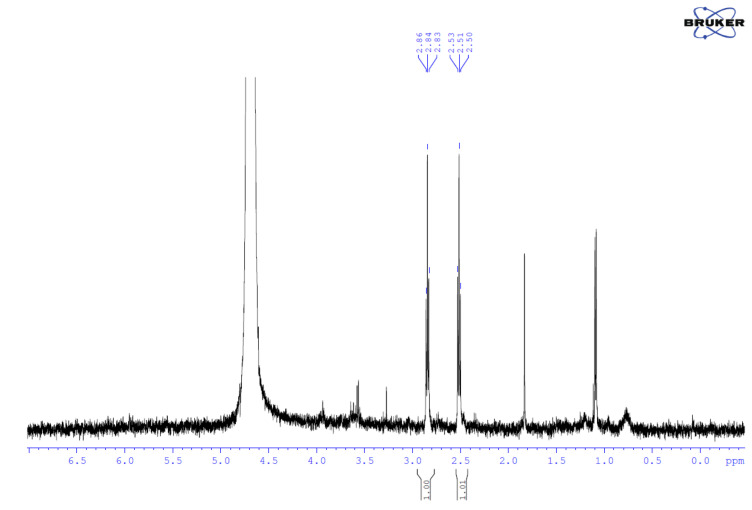
The ^1^HNMR AuNPs-MPA spectrum.

In both cases, NMR measurements were conducted to confirm the identity of the sample—the attachment of the linker (MPA). Pure 3-mercaptopropionic acid gives two triplets in the ^1^H NMR spectrum at chemical shifts of 2.41 and 2.63 ppm. In our samples, we obtained triplets with chemical shifts of 2.51 and 2.84 ppm. Different chemical shifts in these signals indicate that 3-mercaptopropionic acid was attached to Au molecules. An example spectrum is shown in Figure 4. The IR spectra of MPA and AuNPs-MPA are compared in Figure S1. In the FT-IR spectrum of AuNPs-MPA, a characteristic absorption band representing the C = O stretching vibration was observed near 1728 cm^−1^, in contrast to the MPA spectra C = O band near 1700 cm^−1^. The shift in the C = O band and the absence of S-H stretching confirm the interaction of MPA particles with the Au conjugate (Figure S1).

Samples from both syntheses (AuNP-MPAm1 and AuNP-MPAm2) were used for the further synthesis of diosgenin coupling in order to compare the biological properties of the nanoparticles so formed. DG was loaded on AuNPs following the methods described in Section 3.4.3 and Section 3.4.4. MPA contains a -COOH group that can be coupled to the –OH group from DG. During optimization, different types of experiments were used by changing the following parameters: (a) The type of reagent for activation of the carboxyl group. The following carbodiimides were used: DIC (diisopropylcarbodiimide), EDCl (1-(3-dimethylaminopropyl)-3-ethylcarbodiimide hydrochloride), DCC (*N*,*N*′-Dicyclohexylcarbodiimide), and NHS (*N*-hydroxysuccinimide). (b) Use of catalysts: DMAP (4-(Dimethylamino)pyridine), cerium chloride (CeCl_3_). (c) Excess reactants relative to the substrate: 3-fold and 6-fold. (d) The reaction time varied between 24 and 48 h. During optimization, it was observed that the best reactants for the AuNP-MPAm1 substrate were NHS/EDC, and for AuNP-MPAm2, they were DCC with DMAP. A strong absorption band at around 534 nm was observed after coating the AuNPs-MPAm1 surface with DG (Figure 2B, black line). A redshift from 563 to 602 nm was observed, indicating the presence of DG on the AuNPs-MPAm2 surface, which could be associated with a refractive index change of the AuNPs (Figure 2C, black line). The IR spectra of DG and AuNPs-MPA-DG are compared in Figure S2. In the FT-IR spectrum of AuNPs-MPA-DG, characteristic absorption bands present in diosgenin were observed: C-H stretching vibrations (2964–2824 cm^−1^) and C-H deformation vibration (1451 cm^−1^, 1381 cm^−1^). The C = O stretching vibration (1727 cm^−1^) from MPA was present in the FT-IR spectrum of the AuNPs-MPA-DG sample and the -OH stretching vibration (from diosgenin) disappeared in the spectra, confirming the conjugation of diosgenin to AuNPs-MPA (Figure S2).

Two mechanisms have been proposed for the attachment of diosgenin to gold nanoparticles. Method I and Method II of coupling are shown in Figures S3 and S4. The primary reaction products from carbodiimides (1): DCC, DIC, and EDCL with 3-mercaptopropionic acid are adducts (2) of acid and carbodiimide. The adducts react with DMAP to give the acyl pyridinium salt (3), with the type of carbodiimide used being irrelevant. The acyl pyridinium salt reacts with an alcohol (in this case, diosgenin) to form an ester (4). The detailed reaction mechanism of the esterification of alcohols with carbodiimides and DMAP is described in the literature [85]. Activation of the carboxyl group can also be carried out with carbodiimide, which does not leave a difficult-to-solubilize urea derivative after the reaction (just as DCC converts to dicyclohexylurea). Thus, when optimizing the process, DIC (diisopropylcarbodiimide) or EDC (*N*-ethyl-*N*-(3-dimethylaminopropyl)carbodiimide, or EDC hydrochloride and *N*-hydroxysuccinimide or sulfo *N*-hydroxysuccinimide were used.

**Figure 5:**
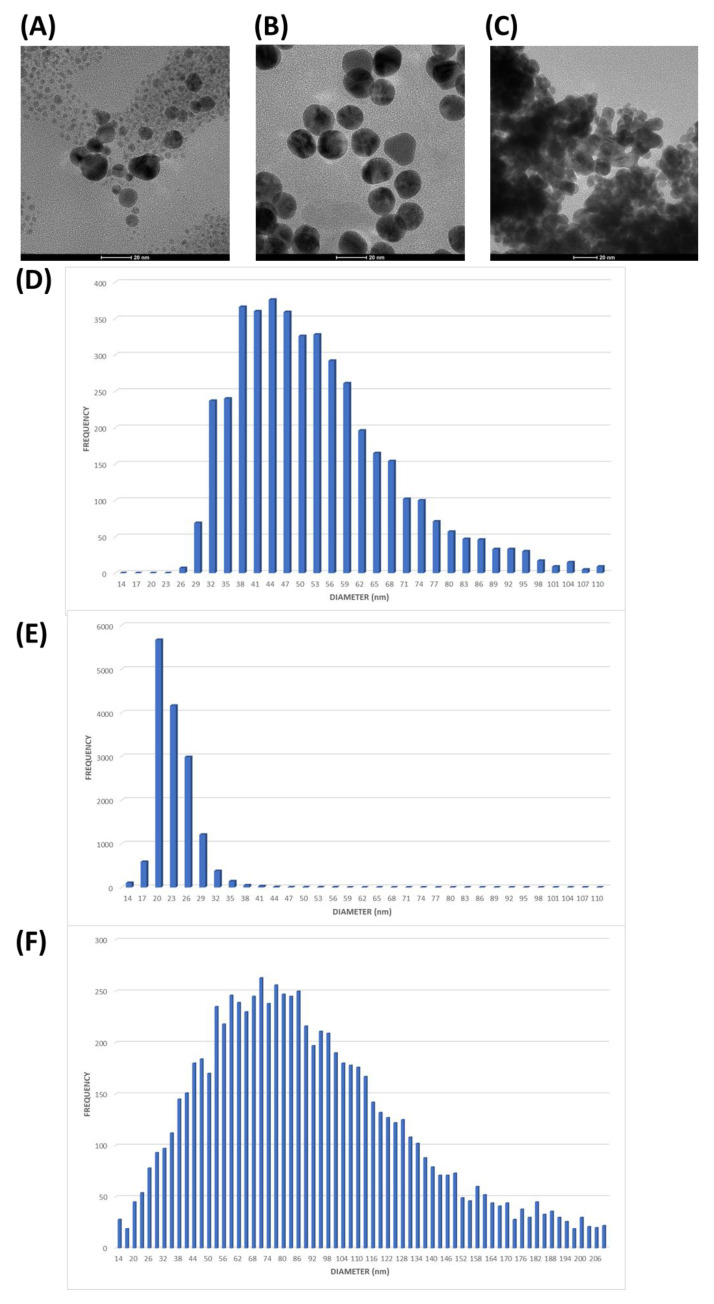
The morphologies of the synthesized conjugates. (**A**–**C**) TEM image of AuNP-MPAm1-DG, AuNP-MPA-TDG, and AuNP-MPAm2-DG, respectively. (**D**–**F**) ICP-MS particle size distribution of AuNP-MPAm1-DG, AuNP-TDG, and AuNP-MPAm2-DG, respectively.

TDG was loaded on AuNPs following the methods described in Section 3.4.2. Figure 5 shows that AuNPs-MPAm1-DG and AuNPs-TDG were well crystallized, spherical in shape, without particle accumulation, and exhibiting an average particle diameter of 53.3 nm and 21.6 nm, respectively. Figure 5 shows that AuNPs-MPAm2-DG exhibited a spherical shape but with particle accumulation and had an average particle diameter of 93.2 nm. This trend was observed as early as the creation stage of AuNPs-MPAm2. It is possible that some nanoparticle accumulation was caused by steric effects and electrostatic forces between DG and AuNPs-MPA surfaces. During the preparation of samples for Transmission Electron Microscopy (TEM), it is plausible that the solvent’s evaporation contributed to nanoparticle accumulation. Therefore, particle sizes are reported from the SP-ICP-MS technique and commented on below.

To characterize the nanoparticle systems used in this work, we used several methods, including Single-Particle ICP-MS (SP-ICP-MS) [86,87,88,89,90,91]. Table 1 summarizes the characteristics of our preparations, including the average and most frequent size (in nm), the concentration of nanoparticles, and gold itself in the nanoparticle and ionic form in the solutions studied. Table 1 clearly shows that the m1 sample leads to the formation of small nanoparticles (most frequent size of 16 nm), with the simultaneous presence of a high concentration of Au in the ionic form (AuNPs-MPAm1). However, considering the specificity of SP-ICP-MS measurements and images obtained in TEM, this concentration can also consist of very small nanoparticles, with a diameter below the detection limit of the method (in our case, it is 13 nm). Sample m2 resulted in the formation of larger nanostructures with a diameter of 31 nm (at the same time, in this case, we no longer observe these very small nanostructures in the solution (Au in ionic form <LOD) (AuNPs-MPAm2)). The attachment of DG to m2 nanoparticles indicates that DG acts as a linker that binds two nanoparticles together, resulting in the formation of stable complexes with a size of 65.5 nm (AuNPs-MPAm2-DG). On the other hand, attaching DG to m1 nanoparticles indicates that this molecule connects three nanoparticles together, creating stable complexes with a diameter of 45 nm (AuNPs-MPAm1-DG). The addition of TDG has a completely different effect on the nanoparticles—here, we do not observe an increase in the size of the nanoparticles, so TDG attaches to individual nanoparticles, and additionally, small structures present in the background disappear (Au in ionic form <LOD) (AuNPs-TDG). Figure 3 and Figure 5 also show that the results obtained in SP-ICP-MS are consistent with those obtained in TEM. The only divergent case is sample AuNPs-MPAm2-DG. The discrepancies in these images are probably due to the evaporation of the solvent in TEM and the formation of polycrystalline forms, while SP-ICP-MS measurements are carried out in an aqueous environment, which protects the nanoparticles from forming aggregates visible as artifacts in TEM.

Table 2 summarizes the ζ-potential of all the products. The ζ-potential of AuNPs delivers information regarding the surface charge of the nanoparticles. The nanoconjugate value of the ζ-potential was found equal to −21.8 mV for AuNPs-TDG, −30.1 mV for AuNPs-MPAm1-DG, and −22.7 mV for AuNPs-MPAm2-DG. All samples have a negative potential charge. The data suggest that all samples demonstrated stability. Consequently, it can be proposed that these nanoparticles will maintain their stability in future in vivo studies following intravenous administration. In general, colloidal solutions were stabilized by DG/MPA loading onto the AuNPs. Therefore, all samples from three different synthesis routes were selected for further biological studies.

The amount of TDG and DG present on the surface of AuNPs, along with the thermal characteristics of the conjugates, was assessed through thermogravimetric analysis. TGA curves are given in SI (Figures S5–S9). The TGA curve of DG shows a significant decrease in the temperature range from 240 to 600 °C; this proves a complete decomposition of DG. The mass loss calculated from the DG curve equals 99.13%. The TGA curve of TDG shows a significant decrease in the temperature range from 200 to 600 °C. It is evidence of the decomposition of TDG. The mass loss calculated from the TDG curve is 97.97%. This information is useful for the contents of DG with the linker and TDG in the conjugates. Results are presented in Table 3.

Smaller nanoparticles have a lower zeta potential (AuNPs-MPAm1) than larger nanoparticles (AuNPs-MPAm2). Therefore, they seem to be more stable. In addition, more diosgenin was attached to AuNPs-MPAm1; therefore, the zeta potential decreased by 39%. In the second case, there was less diosgenin attached to AuNPs-MPAm2; therefore, the zeta potential decreased by 16%. All nanoconjugates with diosgenin in different forms were used to assess the cytotoxic activity for in vitro studies.

### 2.2. In Vitro Viability/Cytotoxicity

The difference between cancer cells (Du-145) and normal prostate cells (PNT-2) lies primarily in their behavior, characteristics, and basic molecular features. The key difference is the occurrence of frequent mutations in cancer cells in genes regulating the cell cycle, apoptosis (programmed cell death), and DNA repair. Most often, changes occur in suppressor genes, such as p53 (a protein responsible for activating DNA repair mechanisms or inducing apoptosis in response to DNA damage). Additionally, they are characterized by an excessive activity of pro-oncogenic pathways, such as PI3K/Akt or NF-κB. These overactive molecular pathways are the site of diosgenin capture, which consequently leads to selective cytotoxic effects only on cancer cells.

Diosgenin holds potential as an antitumor agent for a variety of cancers, including breast cancer, osteosarcoma, colon cancer, leukemia, erythroleukemia, laryngeal cancer, and prostate cancer. Depending on the type of cancer cells, diosgenin may influence various cellular and molecular pathways involved in cancer cell growth, proliferation, survival, and metastasis. One of the mechanisms is the induction of apoptosis by activating the caspase pathway, reducing the expression of antiapoptotic proteins Bcl-2 and increasing the expression of proapoptotic proteins Bax or by disrupting the mitochondrial membrane potential and releasing cytochrome c into the cytosol. Additionally, it can generate ROS, leading to oxidative stress and consequently apoptosis. Diosgenin also affects the proliferation of cancer cells by inhibiting their division (in the G1/S or G2/M phase) by modulating cyclins and cyclin-dependent kinases (CDKs). Additionally, it can disrupt the PI3K/Akt-mTOR signaling pathway, which is key in tumorigenesis.

**Figure 6:**
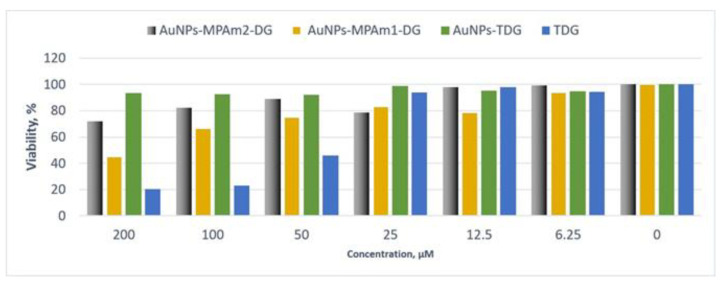
Dependence of the viability (represented in % regarding control) on the concentration of TDG-, DG-, and TDG-conjugated gold nanoparticles incubated with PNT-2 cells for 24 h. The viability was estimated based on the measurement of fluorescence intensity of the converted non-fluorescent resazurin to resorufin in metabolically active cells.

A preliminary study of the influence of thiodiosgenin (TDG), AuNPs-TDG, AuNPs-MPAm2-DG, and AuNPs-MPAm1-DG samples in different concentrations was performed on the Du-145 and PNT-2 cell cultures. The cell viability effect was measured after a 24 h incubation (see Figure 6). The greatest negative impact on the viability of prostate epithelial cells was demonstrated by the thio-derivative of diosgenin in its free form. Compared to the control group (untreated cells), it causes a 54% decrease in viability after the administration of 50 μM. Nevertheless, the obtained results show that the attachment of TDG to gold nanoparticles reduces the negative impact on the viability of prostate epithelial cells. In the case of the AuNPs-TDG sample, no decrease in cell viability is observed even after the administration of the highest concentration. AuNPs-MPAm2-DG and AuNPs-MPAm1-DG systems also have a lower toxicity than TDG against healthy cells. The viability of cells incubated with the highest concentration of the active molecule is as much as 50% and 20% higher compared to TDG.

**Figure 7:**
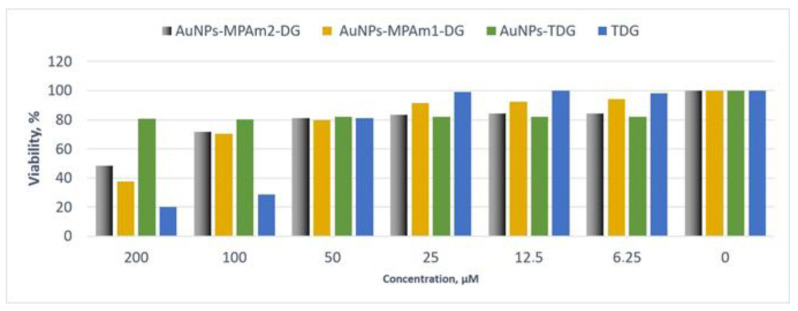
Dependence of the viability (represented in % regarding control) on the concentration of TDG-, TDG-, and DG-conjugated gold nanoparticles incubated with Du-145 cells for 24 h. The viability was estimated based on the measurement of fluorescence intensity of the converted non-fluorescent resazurin to resorufin in metabolically active cells.

The anticancer activity of TDG and the tested conjugates depending on the concentration used is presented in Figure 7. TDG has the greatest impact on the viability of cancer cells. Using a concentration of 100 μM causes a decrease in viability by up to 20%. However, due to its high toxicity towards the normal prostate epithelium, this property cannot be considered successful in modifying the basic DG molecule. Nevertheless, promising results were obtained for DG and TDG conjugates with AuNPs. In the AuNPs-MPAm2-DG and AuNPs-MPAm1-DG samples, a significant decrease in viability was observed at 48.32% and 37.74% for 200 μM. Lin Wang et al. [92] also demonstrated that the exchange of the -OH group with hydroxamic acids and quaternary phosphonium salts increased the antitumor activity compared to diosgenin (against SW620, H358, HCT-116, and Aspc-1 cell lines). However, no results of studies on their potential toxicity were presented. These studies suggest that the introduction of hydrophilic groups in the C-3 position of the A ring, particularly nitrogenous and amide groups, the salt group, hydroxamic acid, etc., enhances pharmacological activity.

In addition, using gold nanoparticles as carriers of the active substances and the new thiol derivative enables the production of multifunctional therapeutic and diagnostic systems. Appropriate functionalization of the AuNP surface allows for the preferential accumulation of AuNPs in cancer cells through the effect of increased permeability and retention (EPR) [93]. Liu et al. modified the AuNP surface using a combination of self-assembled monolayers (SAMs) consisting of a weak electrolyte 11-mercaptoundecanoic acid and a strong electrolyte (10-mercaptodecyl)trimethylammonium bromide and showed that these systems can lead to AuNP aggregation induced by tumor-specific pH, and consequently, increased retention in the tumor matrix and improved tumor internalization [94]. In a physiological environment, no aggregation was observed and the described systems were characterized by appropriate dispersion (particle size of about 20 nm). Huang et al. proposed a different strategy for AuNP modification. The attachment of zwitterionic tetrapeptides allowed for the enzyme-induced (matrix metalloproteinase-9, which is overexpressed in cancer cells) self-assembly of nanoparticles near the cell membrane, which influenced the selection of a size-dependent cellular uptake mechanism [95]. Additionally, AuNPs can absorb near-infrared light, converting it into heat, which leads to the local heating and destruction of cancer cells. Cheng et al. proposed the use of AuNPs decorated with diazirine as an innovative strategy for the aggregation of AuNP in the tumor by photoinduction [96]. Moreover, this modification increased the efficiency of photoacoustic imaging and the efficacy of photodynamic therapy.

The variety of possible surface modifications of AuNPs by attaching active molecules allows using AuNPs not only as a drug carrier but also as a powerful platform in photothermal therapy and tumor imaging. On this basis, the use of AuNPs as a carrier of a diosgenin and a new diosgenin derivative in our studies is very promising and may include not only the delivery of the active molecule to cancer cells but also their destruction by phototherapy and the monitoring of the effectiveness of the treatment by tumor imaging.

## 3. Materials and Methods

### 3.1. Materials—Chemicals and Reagents

The chemicals and reagents used in this study were obtained from POCh (Gliwice, Poland) and Merck KGaA (Darmstadt, Germany): tetrachloroauric (III) acid trihydrate (HAuCl_4_·3H_2_O, 99.0%), sodium borohydride (NaBH_4_, 99.99%), diosgenin (DG, 99.9%), 3-mercaptopropionic acid (MPA, 99.9%), *N*,*N*′-dicyclohexylcarbodiimide for peptide synthesis (DCC, 99.0%), (dimethylamino)pyridine (DMAP, 99.0%), *N*-hydroxysuccinimide (NHS, 98.0%), 1-(3-dimethylaminopropyl)-3-ethylcarbodiimide hydrochloride (EDCl, 99.0%), and *N,N*-dimethylformamide (DMF) ≥ 99.8% for headspace gas chromatography SupraSolv^®^. Dimethyl sulfoxide (DMSO) ≥ 99.8% for gas chromatography was obtained from Supelco (Darmstadt, Germany). Additional materials for SP-ICP-MS: AuNPs—colloidal gold nanoparticles, 30 nm (LGC, Teddington, UK); tuning: NexION Setup Solution 1 µg/L (PerkinElmer Inc., Shelton, CT, USA) and HCl: 30% SUPRAPUR (Merck, Darmstadt, Germany). They were of the highest commercially available purity and were used without prior purification. All solutions were prepared using deoxidized, distilled, and purified water processed through a “Milli-Q” filtration system (Millipore Corporation, Bedford, MA, USA), achieving a final resistance of 18.2 MΩ/cm. TDG was manufactured in Łukasiewicz Research Network—Industrial Chemistry Institute (Łukasiewicz-ICHP), Warsaw, Poland.

### 3.2. Instruments

#### 3.2.1. UV-Vis Spectroscopy Measurements

Ultraviolet–visible spectroscopic analysis was conducted using a UV-Vis Spectrophotometer from the Evolution 220 series by Thermo Scientific (San Jose, CA, USA). The measurements were taken with a 1 cm quartz cell, covering a wavelength range of 190 to 750 nm.

#### 3.2.2. NMR Spectroscopy Measurement

The ^1^H NMR spectra of the studied AuNPs-MPA samples were measured using a Bruker AVANCE III HD spectrometer at the 500 MHz transmitter frequency for 1H. The sample was dissolved in deuterated water (D_2_O). Standard measurement parameters were used with the number of scans equal to 256. The ^1^H NMR spectra were measured at a temperature of 298 K. The residual signal of the solvent was simultaneously the internal standard of the chemical shift (4.70 ppm).

#### 3.2.3. IR Spectroscopy Measurement

ATR-FTIR (attenuated total reflectance–Fourier-transform infrared) spectroscopy was performed using a Nicolet iS50 instrument (Thermo Scientific) with a diamond crystal in the range from 4000 to 600 cm^−1^ and a spectral resolution of 4 cm^−1^. FT-IR spectra were collected from 256 scans. The FT-IR measurements only for AuNPs-MPA were conducted using KBr pellets in transmission mode.

#### 3.2.4. SP-ICP-MS Measurement

All nanoparticle analyses were conducted using NexION 2200 ICP-MS (PerkinElmer Inc., Shelton, CT, USA) with Syngistix Nano App, enabling direct nanoparticle analysis. Samples were measured in triplicate. All the important measurement parameters are shown in Table 4.

Before analysis, the instrument was tuned to achieve the manufacturers’ optimum criteria. The spectrometer was calibrated using an external ionic calibration. The calibration curve was built from 6 points. Standard solutions were prepared at concentrations of 0.01 µg/L, 0.05 µg/L, 0.1 µg/L, 0.5 µg/L, 0.8 µg/L, and 1.0 µg/L, by appropriate dilution of a single-element gold-certified reference material (100 mg/L truQms PerkinElmer) in 1% HCl. The sample flow rate was estimated using the weighting method. Transport efficiency (TE) was calculated using 30 nm AuNPs certified for nanoparticle concentration (LGC, UK). Each of those parameters was checked daily, before starting the measurements. Immediately before measurement, each nanoparticle sample was diluted and measured in SP-ICP-MS mode. After obtaining the result for one dilution, the concentration was confirmed by further dilution—when the nanoparticle number decreased following dilution, the final concentration was calculated.

#### 3.2.5. Morphological Studies

Morphology was assessed byTransmission Electron Microscopy (TEM). The TEM images of the gold nanoparticles modified with MPA, DG, and TDG were generated using a Talos F200X (Thermo Fisher Scientific, Waltham, MA, USA) transmission microscope at 200 kV. The high-angle annular dark-field (HAADF) detector performed the measurements in STEM modes. The specimens for TEM investigations were suspended in ethanol and subjected to an ultrasonic bath; next, a colloid was dropped onto amorphous thin carbon embedded on a Cu grid, where the nanoparticles were left to dry at room temperature.

#### 3.2.6. Electrophoretic Light Scattering (ELS)

The ζ-potentials of AuNPs were measured using a Zetasizer Nano ZS (Malvern Instruments Ltd., Malvern, UK). The angle of the incident light was 173°. Measurements were performed in triplicate and zeta potentials are reported as the mean ± standard deviation (SD).

#### 3.2.7. Thermogravimetry (TGA) Analysis

The TGA measurements were performed by means of a TGA/DSC2 module (Mettler Toledo GmbH, Schwerzenbach, Switzerland). The samples were placed in hermetically sealed 40 μL aluminum pans and perforated before measurements. The samples were heated from 30 to 600 °C at a rate of 10 °C/min under a nitrogen flow of 60 mL/min. The measurements were blank-curve-corrected.

### 3.3. In Vitro Study

Two models of cells, prostate cancer cells Du-145 and normal human prostate PNT-2, were selected for this study. Du-145 cells (ATTC, Bradenton, FL, USA) were grown in Eagle’s Minimum Essential Medium (EMEM) with the addition of 10% (*v*/*v*) fetal bovine serum (FBS). Normal prostate epithelium PNT-2 cells (Sigma Aldrich, Darmstadt, Germany) were maintained in the RPMI medium supplemented with 10% FBS. Cells were cultured in optimal conditions (humidified atmosphere, 37 °C, 5% CO_2_). After the 3rd passage, cells were seeded at a density of 1 × 10^4^ cells per well in a 96-well plate for Du-145 and 5 × 10^3^ for PNT-2. TDG and gold nanoparticles conjugated with DG as AuNPs-MPAm1-DG, AuNPs-MPAm2-DG, and AuNPs-TDG in a DMSO stock solution were diluted in the growth culture medium and added in quintuplicate to the wells in the final concentration (200, 100, 50, 25, 12.5, and 6.25 µM). The maximum content of DMSO was <1%. Both cell lines were incubated with the addition of drugs for 24 h and, after that, the cells were washed in Hank’s Balanced Salt Solution (Gibco, Thermo Fisher Scientific, Waltham, MA, USA) and analyzed by the viability (PrestoBlue™, Thermo Fisher, Waltham, MA, USA).

### 3.4. Methods

#### 3.4.1. Synthesis of Functionalized Gold Nanoparticles (AuNPs) with MPA

AuNPs-MPA were synthesized by a single-phase reduction reaction to avoid the extraction of MPA into the alkaline aqueous phase, similar to that described previously for *p*-mercaptophenol [97]. In detail, hydrogen tetrachloroaurate trihydrate (300 mg, 0.76 mmol) and MPA (157 µL, 1.8 mmol) were dissolved in methanol (150 mL). For the m2 sample: 30 mL of freshly prepared 12 mmol aqueous sodium borohydride was added carefully in a small portion, ca., drop by drop with vigorous stirring. The solution turned black-brown immediately, indicating the formation of gold clusters. For the m1 sample: 30 mL of freshly prepared 6 mmol aqueous sodium borohydride was added carefully in a small portion, ca., drop by drop with vigorous stirring. The solution turned pink immediately, indicating the formation of gold clusters. The reaction solutions m1 and m2 were kept under vigorous stirring at room temperature for 2 h and then cooled in the refrigerator. Subsequently, the solutions were centrifuged three times at 13,000 rpm for 30 min and the precipitate was rinsed with water and methanol between each centrifugation to remove unreacted thiols and by-products. The solvent was removed by centrifugation and dried at a temperature not exceeding 40 °C to give 122 mg of pure product as a dark-brown solid for m2 and 150 mg of pure product as a pink solid for m1. AuNPs-MPA precipitates were powdery; however, they demonstrated very different properties.

#### 3.4.2. Synthesis of Gold Nanoparticles (AuNPs-TDG) by Exchange

Spherical gold nanoparticles (AuNPs) were prepared according to the well-established citrate reduction method by a modified protocol based on the Turkevich method [44]. Subsequently, AuNPs were functionalized with thiodiosgenin (TDG) by exchanging citrate for thiodiosgenin. In detail, a solution of TDG (0.2% *m*/*v*, 5 mL in DMSO) was added to 95 mL of a solution of AuNPs-citr while stirring. The mixture was incubated for 24 h at room temperature and then overnight in the refrigerator. Subsequently, the solution was centrifuged three times at 14,000 rpm for 60 min and the precipitate was rinsed with ethanol between each centrifugation. The mixture was centrifuged to separate the unadsorbed TDG from the conjugate. The solvent was removed by centrifugation and dried without exceeding a temperature of 40 °C. The mass of the precipitate was determined by measuring the solid residue after evaporation of the solvent, which was approximately 21 mg.

#### 3.4.3. Synthesis of Gold Nanoparticles (AuNPs-MPAm2-DG)—DCC/DMAP Coupling Reaction

AuNPs-MPA was employed for the coupling experiments. MPA contains a -COOH group that can be coupled to the –OH group from DG. After prior optimization of the reaction, 80 mg of AuNPs-MPAm2 and 5 mL of DMF were added. To this solution, coupling reagents of DCC (85 mg) and catalyst DMAP (20 mg) and 100 mg of DG were introduced. Then, the reaction mixture was stirred for 24 h at room temperature. The nanoparticles were purified with DMF by decantation/centrifugation (four times, 14,500 rpm, 0.5 h), and then washed with ethanol until free DG in the leachate disappeared and replaced the solvent. The nanoparticles were dried at a temperature of 40 °C for 2 days. The nanoparticles were characterized using a nanozetasizer, UV-Vis, TEM, TGA, FT-IR, and SP-ICP-MS. The obtained yield was >90%.

#### 3.4.4. Synthesis of Gold Nanoparticles (AuNPs-MPAm1-DG NHS/EDCl Coupling Reaction

Under the optimal reaction conditions, to 80 mg of AuNPs-MPAm1, 5 mL of water was added. To this solution, coupling reagents of EDCl in water (100 mg), 100 mg of NHS, and 100 mg of DG in ethanol were introduced. Then, the reaction mixture was stirred for 24 h at room temperature. The particles were purified by decantation/centrifugation (four times, 14,500 rpm, 0.5 h) with water. Then, they were washed with ethanol until free DG in the leachate disappeared and replaced the solvent controlled by UV-Vis. The nanoparticles were dried at a temperature of 40 °C for 2 days. The successful conjugations were monitored by the zeta potential, UV-Vis, TEM, TGA, FT-IR and ICP-MS. The obtained yield was >90%.

## 4. Conclusions

In this article, we focused on the development of the following gold nanoparticles for biological studies: gold nanoparticles (AuNPs) covered by thiodiosgenin (TDG), AuNPs covered by 3-mercaptopropionic acid (MPA) carrying carboxylic groups with different morphology, and diosgenin (DG) linked to AuNPs-MPA. We investigated what molar ratio of sodium borohydride to gold generates small nanoparticles. Conjugates with different morphologies were further conjugated with DG to determine their usefulness in anticancer activities. AuNPs-MPA samples were used for conjugation with DG through carbodiimide chemistry. DG was immobilized on the AuNP surface through an ester bond between the carboxyl and hydroxyl—groups of the MPA residue and DG, respectively. For particle size characterization, we introduced a new technique called SP-ICP-MS. SP-ICP-MS provides quantitative data on nanoparticles by measuring their number, size, and size distribution, as well as the concentration of the ionic form of the element being analyzed in a solution. It is also useful in studies that require determining the dissolution and agglomeration efficiency of nanoparticles. SP-ICP-MS allows for obtaining these key parameters in a single measurement act. This confirms the attractiveness of the SP-ICP-MS technique as a high-throughput characterization of nanoparticles in various samples.

TDG exhibits higher cytotoxic activity towards cancer cells but is not safer for normal prostate epithelial cells (PNT-2) than diosgenin itself. Due to its high toxicity towards a normal prostate epithelium, this property cannot be considered a success in modifying the basic DG molecule. However, it has been proven that the obtained conjugates allow us to reduce the viability of cancer cells while not negatively affecting the viability of normal cells. We have shown the anticancer properties of gold nanoparticles with diosgenin, not only in single NPs but also in polycrystalline structures.

All the obtained conjugates showed antitumor properties; however, for targeted transport, the system referred to as AuNPs-MPAm1-DG is promising, due to the size of the nanoparticles. In conclusion, in the present paper, we report the design of diosgenin nanoconjugates with anticancer properties, showing great potential for further research.

## Figures and Tables

**Table 1:** The characteristics of nanoparticles obtained with SP-ICP-MS.

Sample ID	Most Freq. Size (nm)	Mean Size (nm)	Part. Conc. (parts/mL)	Au in Ionic Form (mg/L)	Au in NPs Form (mg/L)
AuNPs-MPAm1	16 ± 0.35	27.8 ± 0.1	5.82 × 10^12^ ± 7.45 × 10^10^	640 ± 29.8	1264.36 ± 29.84
AuNPs-MPAm2	31.5 ± 0.5	58.85 ± 4.35	4.04 × 10^10^ ± 2.37 × 10^9^	<LOD	83.06 ± 11.46
AuNPs-MPAm2-DG	65.5 ± 1.5	93.15 ± 0.95	4.23 × 10^10^ ± 5.44 × 10^8^	2.5 ± 0.06	345.85 ± 6.14
AuNPs-TDG	15.5 ± 0.5	21.6 ± 1.1	3.81 × 10^12^ ± 6.80 × 10^11^	<LOD	381.31 ± 10.44
AuNPs-MPAm1-DG	45 ± 0.11	53.25 ± 0.51	1.70 × 10^11^ ± 1.66 × 10^9^	31.43 ± 4.04	290.51 ± 2.5

**Table 2:** The characteristics of nanoparticles obtained with ELS.

Samples	ζ-Potential	s (Standard Deviation)
AuNPs-MPAm1	−52.3	0.33
AuNPs-MPAm2	−27.0	0.36
AuNPs-TDG	−21.8	0.51
AuNPs-MPAm1-DG	−30.1	0.31
AuNPs-MPAm2-DG	−22.7	0.50

**Table 3:** Results of TGA analysis.

Samples	TGA, Δ m, [%] (m/m)
AuNPs-TDG	−6.38
AuNPs-MPAm1-DG	−27.61
AuNPs-MPAm2-DG	−3.47

**Table 4:** SP-ICP-MS measurements of experimental parameters.

Parameter		Characteristics	
		Unit	SP-ICP-MS
Spray chamber		Quartz cyclonic	
Nebulizer		Meinhard concentric	
Torch		Quartz	
Injector	Material	Quartz	
	I.D.	mm	2.0
Sampler, skimmer, hyper skimmer with OmniRing		Ni	
Generator	Frequency	MHz	34
	Power	W	1600
Plasma gas flow		L/min	15
Auxiliary gas flow		L/min	1.2
Nebulizer gas flow *		L/min	0.98–1.04
UCT Measurement mode			STD
UCT gas flow		mL/min	0
Measured isotopes			^197^Au
Peristaltic pump	Numbers of rollers	12	
	Turns	rpm	−35
Sample tube	Material, marks	PVC, Orange/Green	
	I.D.	mm	0.38
Waste tube	Material, marks	Santoprene, Gray/Gray	
	I.D.	mm	1.3
Sample flow rate *		mL/min	0.19–0.24
Dwell time		µs	50
Transport efficiency *		%	7.8–8.6

* Parameter optimized daily.

## Data Availability

Data are contained within the article and Supplementary Materials.

## Supplementary Materials

The following supporting information can be downloaded at: https://www.mdpi.com/article/10.3390/ijms26031088/s1.
