# Supplementary material for: Preparation of Diosgenin-Functionalized Gold Nanoparticles: From Synthesis to Antitumor Activities
Source: Int J Mol Sci. 2025 Jan 27;26(3):1088. doi: 10.3390/ijms26031088 (PMC11817374; doi:10.3390/ijms26031088)
Supplement: Supplementary file 1 [file ijms-26-01088-s001.zip › ijms-3390491-supplementary.pdf]

Supporting Information

Preparation of Diosgenin-Functionalized Gold Nanoparticles. From synthesis to antitumor activities.

Elżbieta U. Stolarczyk[1], Weronika Strzempek[2], Magdalena Muszyńska[3], Marek Kubiszewski[4], Anna B. Witkowska[1], Kinga Trzcińska[4], Piotr Wojdasiewicz[5], and Krzysztof Stolarczyk [3,*]

[1] Spectrometric Methods Department, National Medicine Institute, 30/34 Chełmska Street, 00-725 Warsaw, Poland e.stolarczyk@nil.gov.pl (E.U.S.); anna.witkowska@wum.edu.pl (A.B.W.)

[2] Faculty of Chemistry, Jagiellonian University, 2 Gronostajowa Street, 30-387 Krakow, Poland; weronika.skuza.s@gmail.com (W.S.)

[3] Faculty of Chemistry, University of Warsaw, 1 Pasteura Street, 02-093 Warsaw, Poland, m.muszynska@chem.uw.edu.pl (M.M.); kstolar@chem.uw.edu.pl (K.S.)

[4] Analytical Research Section, Pharmaceutical Analysis Laboratory Łukasiewicz Research Network – Industrial Chemistry Institute, 8 Rydygiera Street, 01-793 Warsaw, Poland; marek.kubiszewski@ichp.lukasiewicz.gov.pl (M.K.); kinga.trzcinska@ichp.lukasiewicz.gov.pl (K.T.)

[5] Department of Biophysics, Physiology and Pathophysiology, Faculty of Health Sciences, Medical University of Warsaw, Chałubińskiego 5, 02-004 Warsaw, Poland; piotr.wojdasiewicz@wum.edu.pl (P.W.)

*Correspondence: kstolar@chem.uw.edu.pl

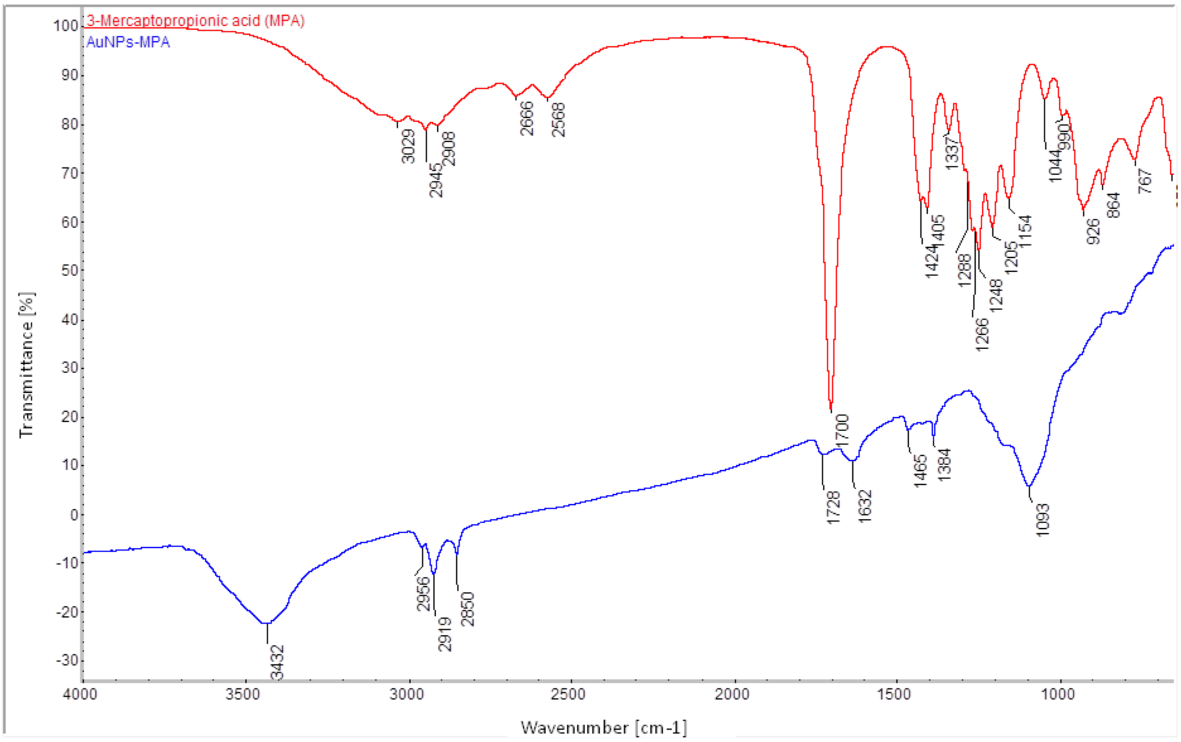

Figure S1. A comparison of the IR spectra of MPA and AuNPs-MPA.

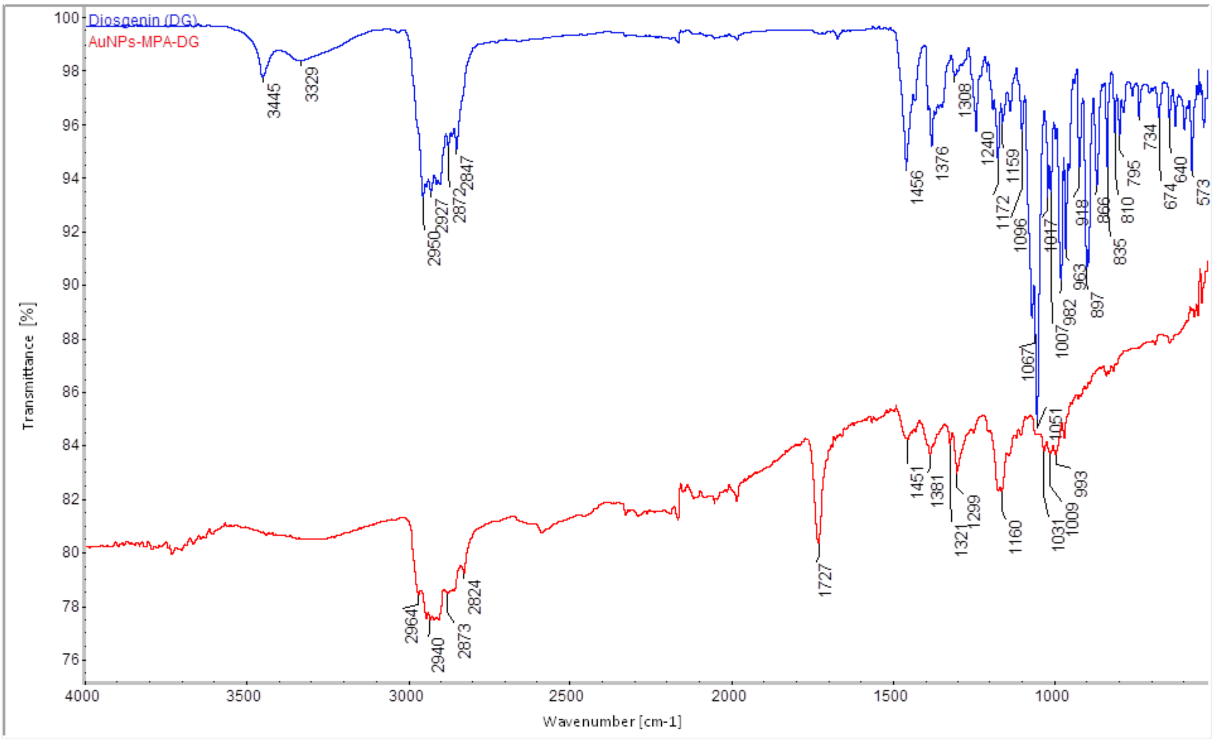

Figure S2. A comparison of the IR spectra of diosgenin (DG) and AuNPs-MPA-DG.

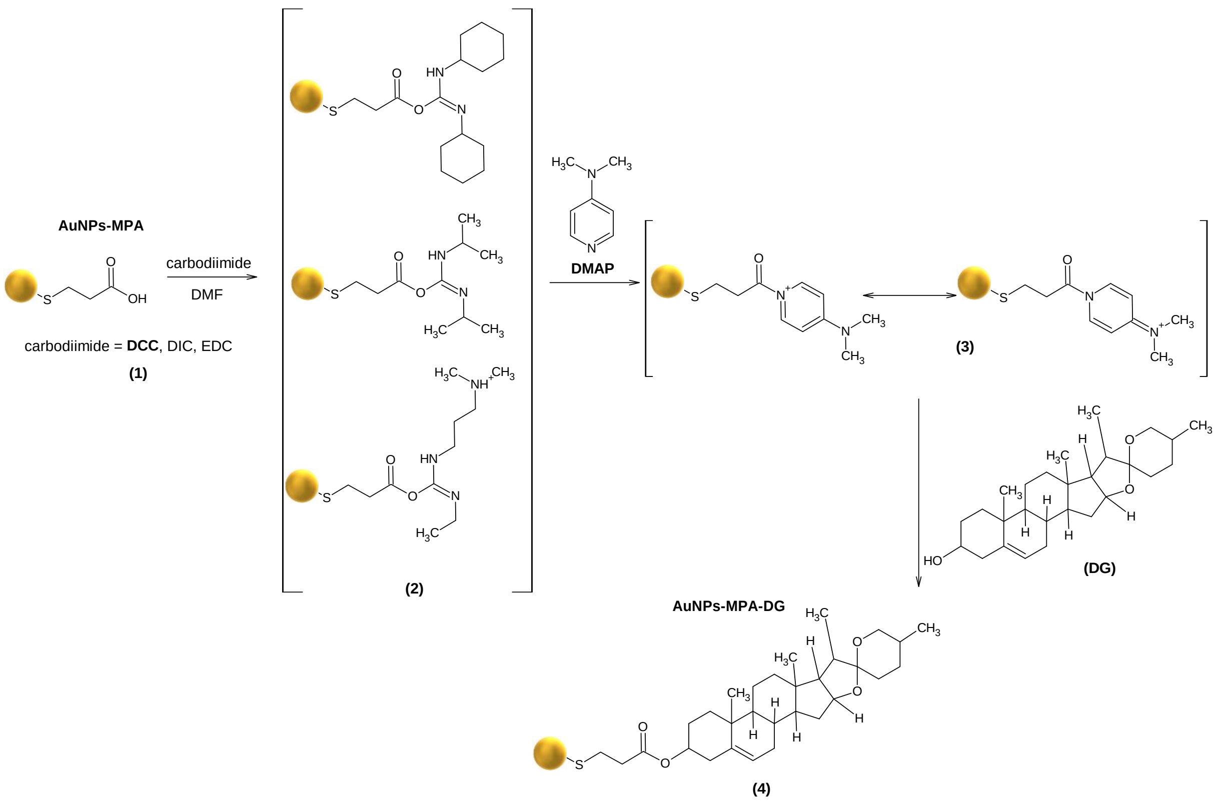

Figure S3. Method I used in the coupling of diosgenin to gold nanoparticles for AuNPs-MPAm2-DG.

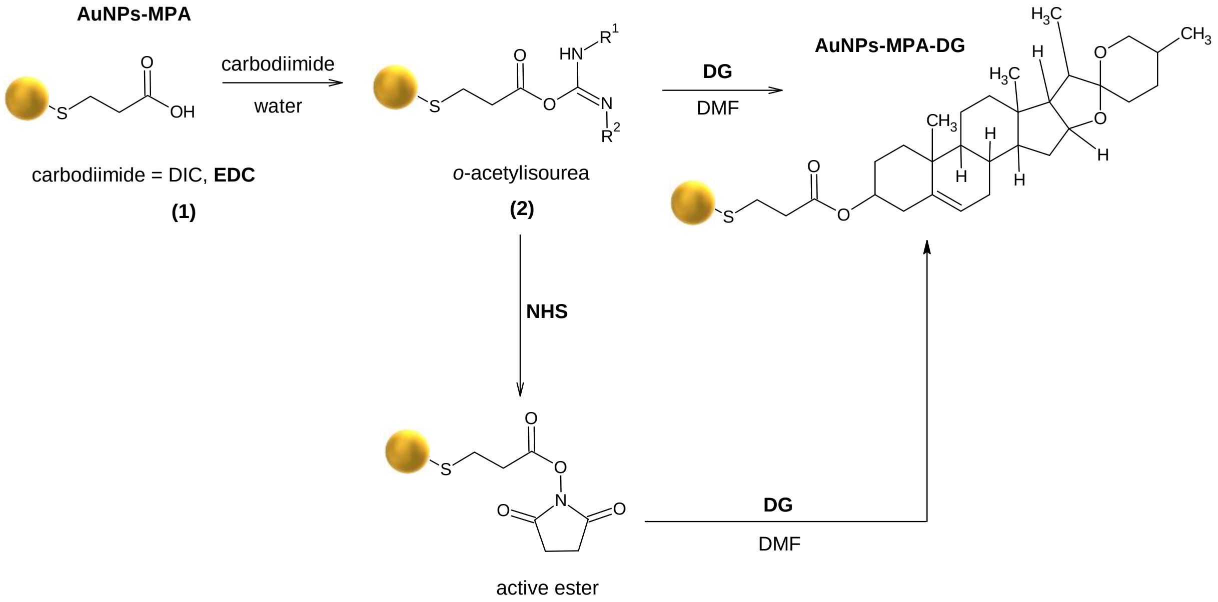

Figure S4. Method II used in the coupling of diosgenin to gold nanoparticles for AuNPs-MPAm1-DG.

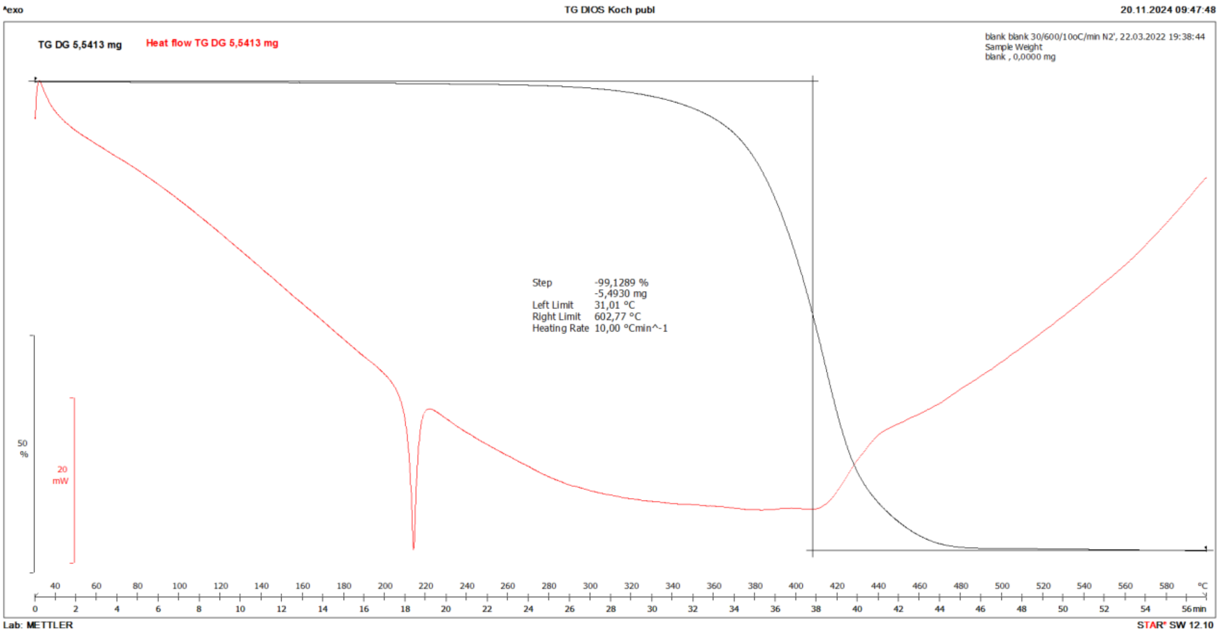

Figure S5. TGA curves of DG.

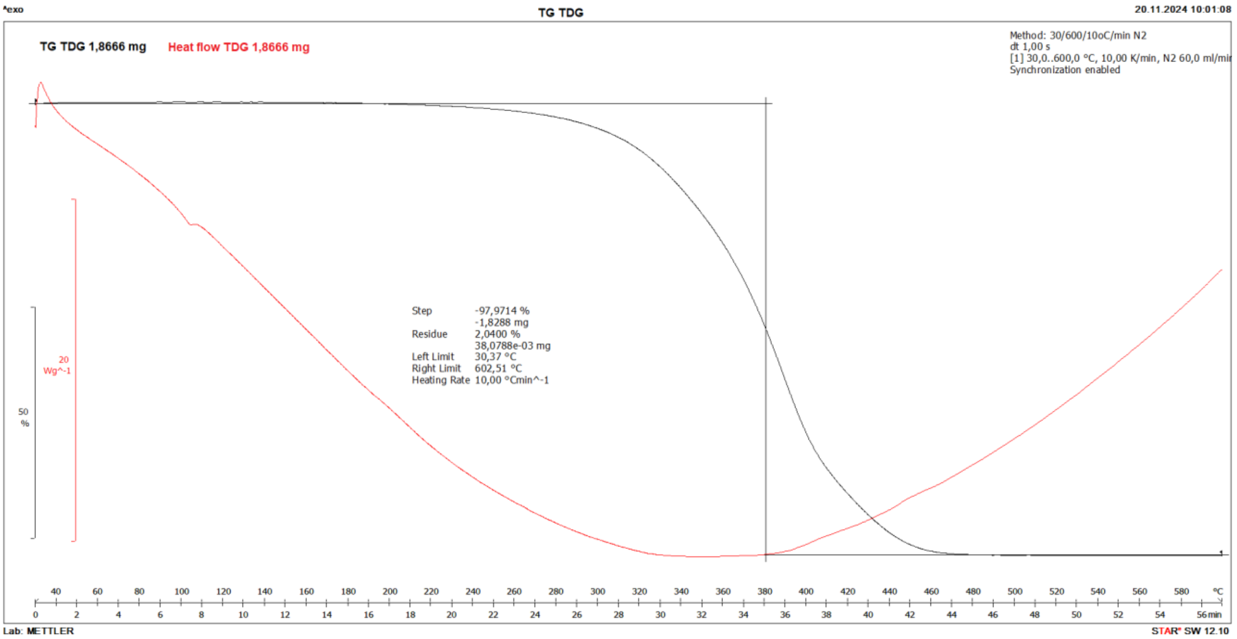

Figure S6. TGA curves of TDG.

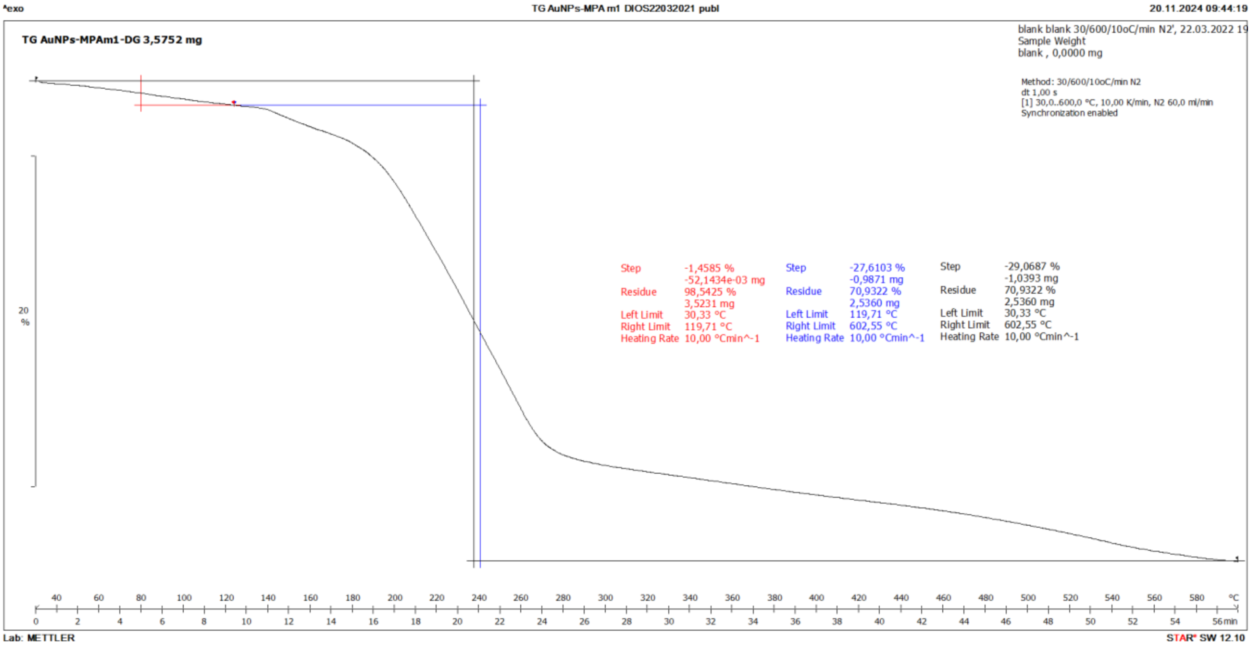

Figure S7. TGA curves of AuNPs-MPA m1-DG.

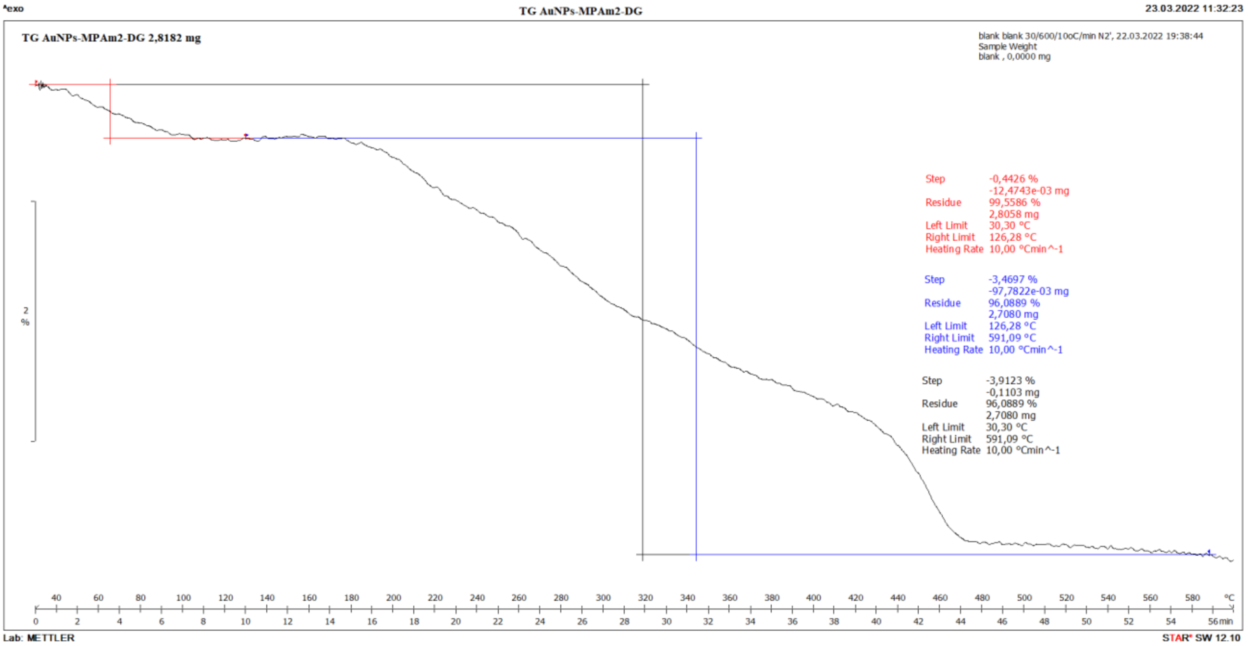

Figure S8. TGA curves of AuNPs-MPAm2-DG.

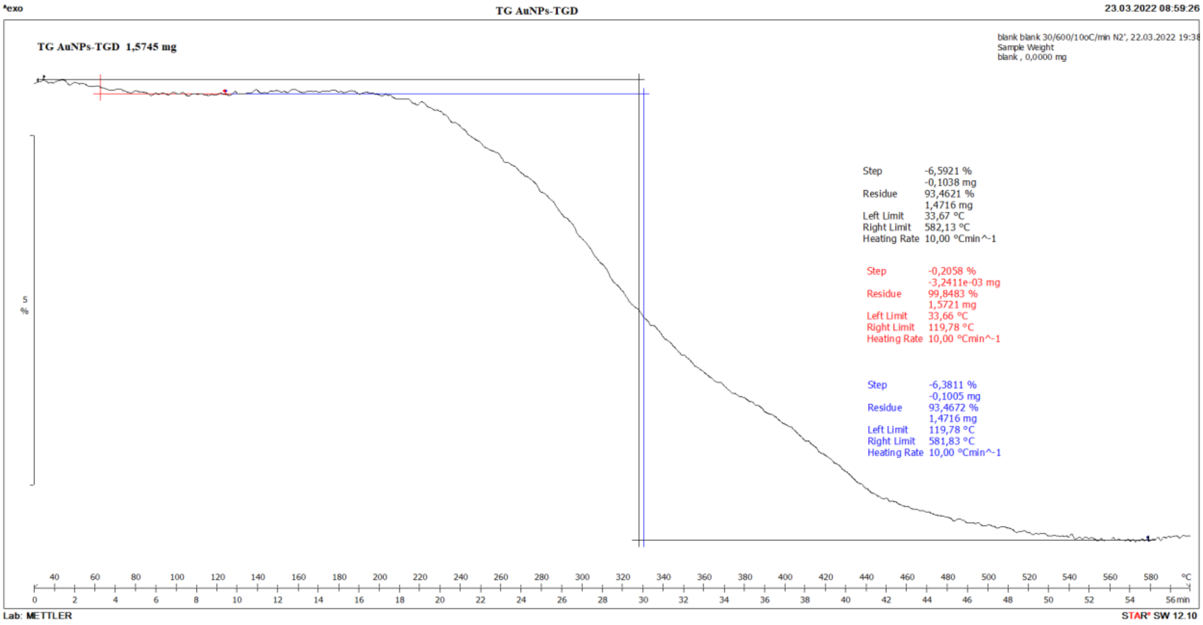

Figure S9. TGA curves of AuNPs-TDG.
